# Influence of Acute Water Ingestion and Prolonged Standing on Raw Bioimpedance and Subsequent Body Fluid and Composition Estimates

**DOI:** 10.2478/joeb-2022-0003

**Published:** 2022-05-20

**Authors:** Grant M. Tinsley, Matthew T. Stratton, Patrick S. Harty, Abegale D. Williams, Sarah J. White, Christian Rodriguez, Jacob R. Dellinger, Baylor A. Johnson, Robert W. Smith, Eric T. Trexler

**Affiliations:** 1 Energy Balance & Body Composition Laboratory; Department of Kinesiology & Sport Management, Texas Tech University, Lubbock, TX, USA; 2 Department of Nutrition and Health Sciences, University of Nebraska-Lincoln, Lincoln, Nebraska, USA; 3 Stronger By Science LLC Raleigh, NC, USA

**Keywords:** Bioelectrical impedance analysis, hydration, body composition, body fat, muscle mass

## Abstract

This study evaluated the influence of acute water ingestion and maintaining an upright posture on raw bioimpedance and subsequent estimates of body fluids and composition. Twenty healthy adults participated in a randomized crossover study. In both conditions, an overnight food and fluid fast was followed by an initial multi-frequency bioimpedance assessment (InBody 770). Participants then ingested 11 mL/kg of water (water condition) or did not (control condition) during a 5-minute period. Thereafter, bioimpedance assessments were performed every 10 minutes for one hour with participants remaining upright throughout. Linear mixed effects models were used to examine the influence of condition and time on raw bioimpedance, body fluids, and body composition. Water consumption increased impedance of the arms but not trunk or legs. However, drift in leg impedance was observed, with decreasing values over time in both conditions. No effects of condition on body fluids were detected, but total body water and intracellular water decreased by ~0.5 kg over time in both conditions. Correspondingly, lean body mass did not differ between conditions but decreased over the measurement duration. The increase in body mass in the water condition was detected exclusively as fat mass, with final fat mass values ~1.3 kg higher than baseline and also higher than the control condition. Acute water ingestion and prolonged standing exert practically meaningful effects on relevant bioimpedance variables quantified by a modern, vertical multi-frequency analyzer. These findings have implications for pre-assessment standardization, methodological reporting, and interpretation of assessments.

## Introduction

Bioimpedance technology has been widely used to monitor a variety of biological components, including vascular function, hydration status and fluid accumulation, disease prognosis, wound healing, and body composition [1]. While physiological applications vary, bioimpedance technologies rely on core biophysical principles, such as the application of Ohm’s Law to describe the relationship between voltage changes, the flow of electrical current, and electrical impedance (Z) [1]. Z is composed of two primary components, resistance (R) and reactance (Xc). While R quantifies the opposition to current flow, Xc represents the capacitive properties of the cells and tissues [2]. R and Xc can also serve as input values for the calculation of phase angle (ϕ), a well-established clinical parameter. ϕ is frequently presented as a noninvasive metric of cellular health, membrane integrity, and the quantity and quality of soft tissue. Furthermore, numerous investigations have reported the potential utility of ϕ as a prognostic indicator of nutritional status, physiological function, and mortality [3, 4]. Despite a resurgent interest in the utilization of raw bioelectrical parameters, including ϕ [1, 5], the estimation of body fluids and body composition remain two frequent applications of bioimpedance technology [6, 7].

As with many methods of body composition assessment, pre-assessment standardization is critical for reducing biological error and improving the utility of output data [2, 8, 9]. Abstention from eating, exercise, moderate or vigorous physical activity, and medication or substance ingestion are common components of pre-assessment standardization. Several investigations have indicated that transient alterations of bioimpedance-derived body composition estimates are observed in response to acute food and fluid intake [10, 11, 12, 13]. Importantly, the magnitudes of these errors may be large enough to obfuscate true changes in body composition over time [14, 15]. Although it is generally believed that bioimpedance technologies may be particularly susceptible to errors caused by transitory alterations in hydration, many investigations utilizing bioimpedance technology either do not report a mandatory abstention from fluid ingestion prior to assessment or utilize durations of fluid abstention shorter than an overnight period. Limited direct information is available to inform the choice of fluid restriction or allowance prior to bioimpedance assessments, specifically whether it is preferable to implement an overnight dry fast (i.e., no foods or fluids ingested for ≥8 hours) as compared to an overnight fast that allows either ad libitum or prescribed water intake (i.e., water fasting). While preliminary information is available for select single-frequency bioelectrical impedance analysis (BIA) analyzers [16, 17], most contemporary devices remain unexamined.

In addition to questions regarding standardization of fluid ingestion in the hours preceding bioimpedance assessments, other methodological questions remain. The influence of body posture is particularly relevant due to current utilization of both traditional analyzers requiring a supine body position and newer vertical analyzers requiring an upright (i.e., standing) body position. Through radionuclide dilution techniques, it has been established that plasma volume decreases when standing relative to seated and supine body positions, largely due to fluid movement into the interstitial space [18]. While several reports have clarified the time course of bioelectrical responses to postural changes, these investigations have primarily focused on transitioning from standing to the supine position due to the traditional implementation of supine bioimpedance assessments [19, 20, 21, 22]. To our knowledge, investigations reporting serial bioimpedance assessments have not employed standing durations longer than 30 minutes nor used modern vertical bioimpedance analyzers [20, 22, 23, 24].

Based on these important methodological considerations, the purpose of the present investigation was to determine the effects of acute water ingestion on changes in raw bioelectrical variables and subsequent estimates of fluid compartments and body composition variables. Additionally, the present design allowed for examination of changes in these variables in response to maintaining the upright body position for a longer duration than previous investigations.

## Materials and methods

### Overview and Participants

This study was a randomized crossover trial. Each participant completed two conditions: 1) serial BIA assessments following acute water ingestion (water condition), and 2) serial BIA assessments with no water ingestion (control condition). Individuals were eligible for participation if they were between the ages of 18 and 40; generally healthy; participated in exercise, sport, or a physically demanding job at least weekly; did not smoke; and were weight stable, defined as no change in body mass (BM) greater than 2.3 kg in the previous month. Individuals were ineligible if they were a bodybuilder or similar athlete due to potential concerns of bioimpedance estimates in these populations [25], or if they had a pacemaker or other implanted electrical device. All participants read and signed a university-approved consent document prior to participation. Twenty participants consented and completed the study. Following consent, each participant was assigned to complete the water and control conditions using sequences produced from a random sequence generator (http://www.random.org).

### Laboratory Procedures

Upon reporting to the laboratory, participants were asked to void their bladders and provide a urine sample for subsequent assessment of urine specific gravity (USG). Thereafter, a baseline BIA assessment was performed, followed by a 5-minute period during which participants consumed water (water condition) or stood quietly (control condition). The dose of bottled water consumed during the W condition was 11 mL/kg body mass. This relative dose corresponded to an absolute intake of (mean ± SD) 807 ± 225 mL, with a range of 531 to 1360 mL. Beginning 10 minutes after this time period, BIA assessments were performed every 10 minutes for one hour (i.e., at 10, 20, 30, 40, 50, and 60 minutes after the 5-minute water consumption period). After each BIA assessment, participants stepped off the analyzer and remained in the upright position adjacent to the analyzer. Participants remained standing throughout the entire visit for both conditions. The selection of total assessment duration was based on the rapid absorption of ingested water following an overnight fast [26].

After the final BIA assessment, participants were asked to provide an additional urine sample. The USG of urine samples was assessed via digital refractometer (PA201X-093, Misco, Solon, OH, USA). After a short washout period of one to eight days (mean ± SD: 3.5 ± 2.8 days), each participant completed the alternate condition. Scheduling of visits occurred without consideration of the menstrual cycle due to the short duration between visits, utilization of baseline assessments in each condition, the frequent lack of regular cycles in our participant pool of active females [27], and prior research indicating minimal influence of the menstrual cycle on bioimpedance parameters [28, 29, 30].

### Bioelectrical Impedance Analysis

BIA assessments were performed using the InBody 770 direct segmental multi-frequency analyzer (InBody, Seoul, South Korea). This analyzer contains eight electrodes, with four placed in contact with the bottom of the feet (two at each heel and front sole) and four placed in contact with the hands (two at each thumb and palm). Assessments are conducted in the standing position, with the shoulder abducted and arms straightened to ensure no contact between the arms and torso. The analyzer uses six measurement frequencies ranging from 1 to 1000 kHz (i.e., 1, 5, 50, 250, 500, and 1000 kHz) with an applied current of 80 μA (±10 μA). Z at all six measurement frequencies is reported by the device, along with Xc values at 5, 50, and 250 kHz and 50 kHz ϕ values.

Segmental and whole-body measurements are made for all bioelectrical variables through the analyzer’s direct segmental measurement technology. Data from multiple pairs of electrodes are integrated to determine voltage in common loops and allow for subsequent segment specific values to be generated [31]. Z is established based on Ohm’s law, R through Cole modeling, Xc in relation to the phase delay measured by the device, and ϕ as the angle between the R and Xc vectors [2]. The technical error of the measurement (TEM) for whole-body bioimpedance variables at 50 kHz in our laboratory was 2.4 Ω for R and Z, 0.4 Ω for Xc, and 0.03 ° for ϕ For body fluids, ECW is estimated based on Z at low measurement frequencies, TBW is estimated based on Z at high measurement frequencies, and ICW represents the difference between TBW and ECW. Fluids are estimated for each segment using that particular segment’s bioimpedance values, with the head, neck, hands, and feet intentionally estimated due to known issues with current penetration or disproportionately high Z. Our laboratory’s TEM values for TBW, ECW, and ICW were 0.02 kg, 0.00 kg, and 0.02 kg, respectively. To estimate LBM from TBW, the analyzer uses a proprietary algorithm rather than the commonly assumed ~73.3% water content. Our laboratory’s TEM values were 0.14%, 0.05 kg, and 0.03 kg for percent body fat (%BF), lean body mass (LBM), and fat mass (FM), respectively.

A scale is integrated with the BIA analyzer and automatically determined BM before commencing the bioimpedance measurements. Height was assessed using a mechanical stadiometer (Seca GmbH & Co., Hamburg, Germany), to the nearest 0.1 cm, and was manually entered into the analyzer’s software interface. Total and segmental outputs of raw bioelectrical variables, body fluids, and body composition were analyzed in the present investigation. The manufacturer-provided term of “lean body mass” (LBM) is utilized in the present report, for the sake of clarity, although inspection of results confirms this entity is equivalent to fat-free mass (i.e., BM minus FM).

### Data Analysis

The purpose of the analysis was to evaluate the effects of fluid intake on the full range of variables quantified by the BIA device. The sample size was selected based on a prior investigation indicating alteration of body composition estimates after acute water intake [16], as well as practical constraints. The prevalence of missing data was 0.36% for each bioimpedance variable due to failure to save the output from a single BIA test in one condition. Data from the adjacent time points within the same condition were averaged to replace these values. The prevalence of missing data for USG was 12.5% due to inability of some participants to provide urine samples. Multiple imputation with 100 iterations was performed using the *mice* software package [32] in order to replace the missing values due to lack of relevant adjacent data points. Data were analyzed using linear mixed-effects models with a random intercept for participant. A first-order autoregressive (AR1) variance-covariance matrix was used with the correlation form of 𝑇𝑖𝑚𝑒|𝑃𝑎𝑟𝑡𝑖𝑐𝑖𝑝𝑎𝑛𝑡/𝐶𝑜𝑛𝑑𝑖𝑡𝑖𝑜𝑛. In all models, the reference groups were control (i.e., no water consumption) for condition and the baseline assessment for time. Models were implemented using the *nlme* package for R [33, 34] and were fit by maximizing the restricted log-likelihood (REML). Fixed effects of time, condition, and the time by condition interaction were examined using joint tests from the *emmeans* software package [35]. Model estimates (i.e., *b* and associated 95% confidence intervals [CI]) were evaluated and visualized using the *sjPlot* R package [36]. Line plots with within-subjects error bars [37, 38] were also generated for each outcome.

Due to the exploratory nature of the analysis, results for all variables of potential interest quantified by the BIA device are presented within the main manuscript or supplementary materials. To control the familywise error rate, a Bonferroni correction was manually applied to the traditional alpha level of 0.05. Therefore, statistical significance was accepted at *p*<0.00057 (0.05/87 outcomes) for effects of condition and time, as well as condition by time interactions. Evaluation of the statistical significance of *b* estimates for terms within linear mixed effects models implemented the cutoffs used within the *sjPlot* package as indicated in figure legends accompanying these plots.

### Informed consent

Informed consent has been obtained from all individuals included in this study.

### Ethical approval

The research related to human use has been complied with all relevant national regulations, institutional policies and in accordance with the tenets of the Helsinki Declaration, and has been approved by the authors’ institutional review board or equivalent committee (protocol ID: IRB2019-729).

## Results

### Body Mass and USG

**Fig.1:**
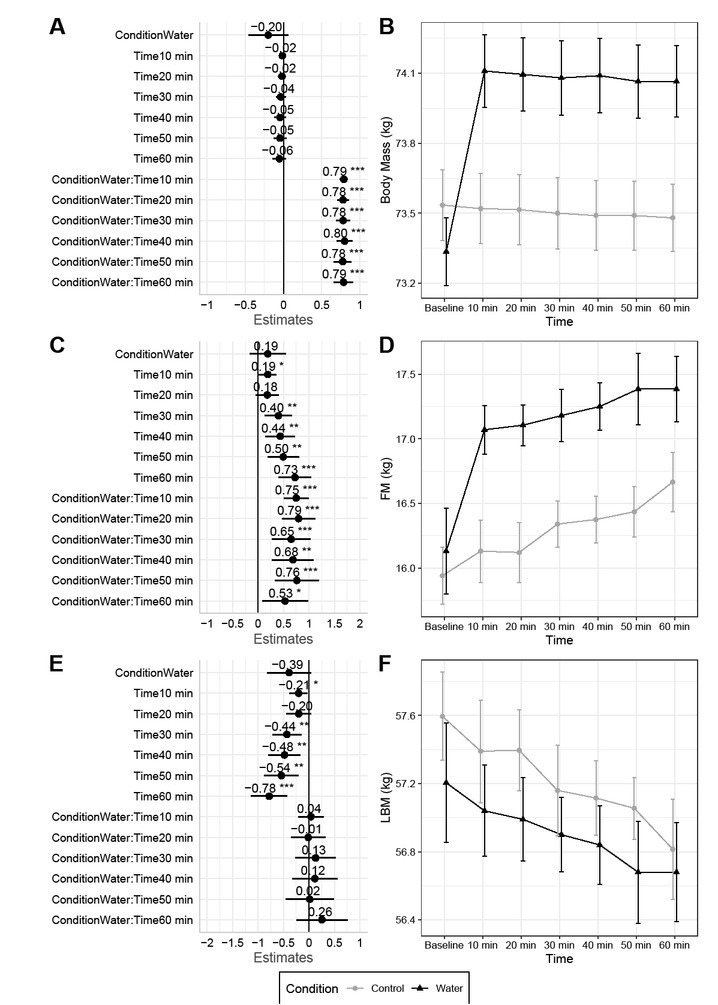
Body Mass and Total Body Composition. Changes between baseline and subsequent time points are displayed for body mass (A – B), fat mass (C – D), and lean body mass (E – F). Linear mixed model coefficients (*b*) and their 95% confidence intervals are displayed in panels A, C, and E. For all models, the reference groups were control (i.e., no water consumption) for condition and the baseline assessment for time. * indicates *p* ≤ 0.05; ** indicates *p* ≤ 0.01; *** indicates *p* ≤ 0.001.

Twenty participants completed the study protocol (Table 1). Upon water consumption in the water condition, BM immediately increased by ~0.8 kg on average and remained stable thereafter (*p*<0.0001 for condition×time interaction; Figure 1A – 1B). A condition ×time interaction was also present for USG (*p*=0.0004; Supplementary Figure 1), which indicated that USG decreased from pre to post in the water condition but not the control. *P*-values for all outcomes are displayed in the Appendix.

**Table 1:** Participant characteristics. Data presented as mean ± SD.

	All (n=20)	Females (n=10)	Males (n=10)
**Age (y)**	22.2 ± 2.7	21.1 ± 1.6	23.2 ± 3.3
**Height (cm)**	172.9 ± 10.4	166.2 ± 6.1	179.6 ± 9.5
**Weight (kg)**	73.3 ± 20.4	59.0 ± 8.4	87.6 ± 18.8
**BMI (kg/m^2^)**	24.1 ± 4.3	21.3 ± 2.3	27.0 ± 4.1
**Body fat (%)**	21.8 ± 5.5	23.0 ± 4.0	20.5 ± 6.6

### Raw Bioimpedance

Select differences in Z, Xc, and ϕ were observed between conditions. Condition by time interactions indicated that Z values for the arms increased over time to a greater extent in the water condition as compared to control for lower measurement frequencies of 1, 5, and 50 kHz (Figure 2A, 2B; Supplementary Figures 2 – 6). At higher frequencies of 250 – 1000 kHz, most *p* values were low but higher than the Bonferroni-adjusted significance level (i.e., *p-*values of 0.01 to 0.001) (Supplementary Figures 7 – 12). In contrast to the arms, no statistically significant condition by time interactions were observed for Z values of the trunk and legs at any measurement frequency (Figure 2C – 2F; Supplementary Figures 13 – 28). However, drift in leg Z was observed in both conditions, with decreasing values over the measurement duration in both conditions (*p*<0.0001 for time main effects at all measurement frequencies). In contrast, no significant time main effects were observed for trunk Z.

**Fig.2:**
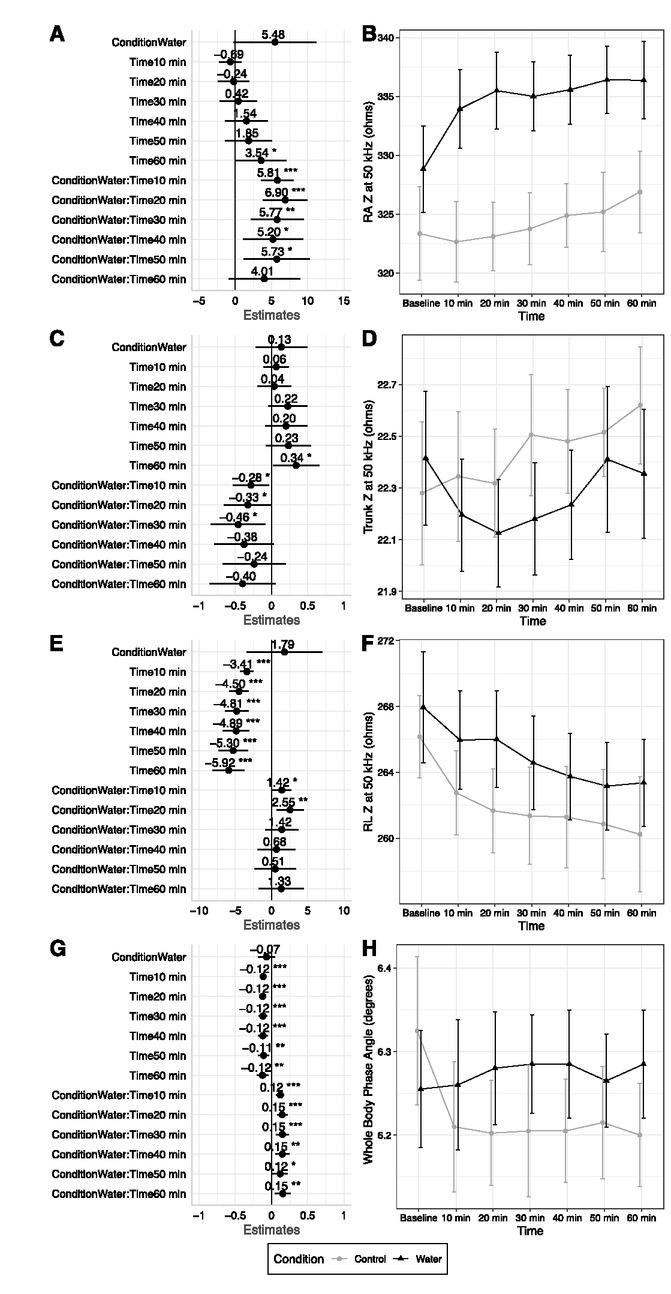
Raw Bioimpedance at 50 kHz. Changes between baseline and subsequent time points are displayed for right arm impedance at the 50 kHz measurement frequency (A – B), trunk impedance at the 50 kHz measurement frequency (C – D), right leg impedance at the 50 kHz measurement frequency (E – F), and whole body phase angle at the 50 kHz frequency (G – H). Linear mixed model coefficients (*b*) and their 95% confidence intervals are displayed in panels A, C, E, and G. For all models, the reference groups were control (i.e., no water consumption) for condition and the baseline assessment for time. * indicates *p* ≤ 0.05; ** indicates *p* ≤ 0.01; *** indicates *p* ≤ 0.001.

Similar to Z, condition by time interactions indicated Xc values for the arms increased over time to a greater extent in the water condition as compared to control, particularly at the 5 kHz frequency (Supplementary Figures 29 – 34).

While no differences in leg Xc were observed due to water consumption, Xc values decreased over time at all frequencies (*p*<0.0001 for time main effects; Supplementary Figures 35 – 40). For trunk Xc, the only significant effect was a time main effect (*p*=0.0004) indicating increased Xc values over time at the 5 kHz measurement frequency (Supplementary Figures 41 – 43).

**Fig.3:**
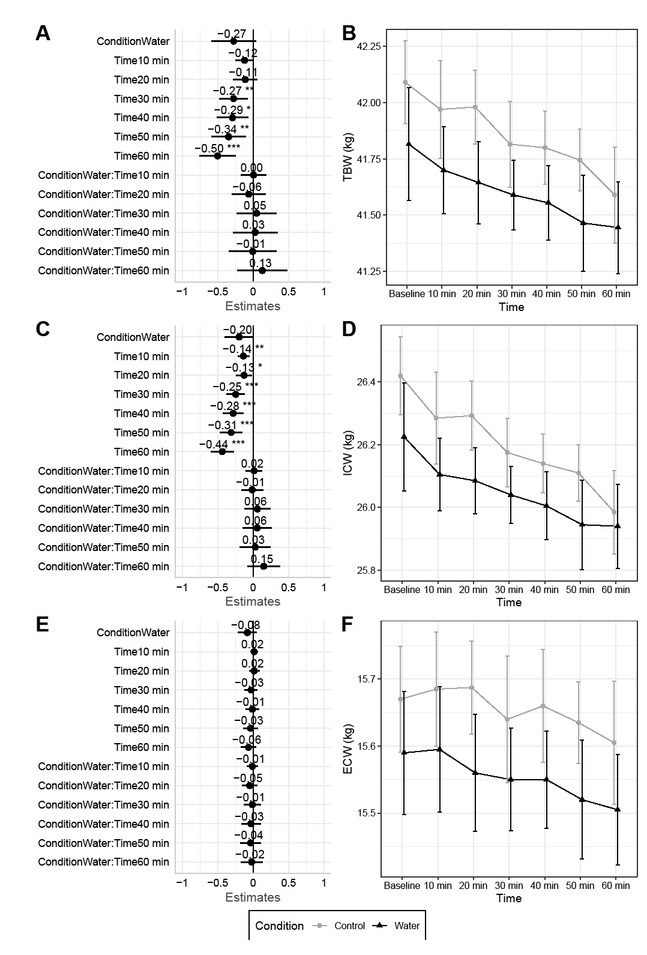
Body Fluids. Changes between baseline and subsequent time points are displayed for total body water (A – B), intracellular water (C – D), and extracellular water (E – F). Linear mixed model coefficients (*b*) and their 95% confidence intervals are displayed in panels A, C, and E. For all models, the reference groups were control (i.e., no water consumption) for condition and the baseline assessment for time. * indicates *p* ≤ 0.05; ** indicates *p* ≤ 0.01; *** indicates *p* ≤ 0.001.

A condition by time interaction was present for whole body ϕ (*p*=0.0004; Figure 2G – 2H). Mean whole body ϕ values remained stable within a range of 6.26º to 6.29º in the water condition. In the control condition, ϕ values decreased from 6.32º to 6.21º immediately following the baseline time point and remained in the range of 6.20º to 6.21º thereafter. ϕ of the arms and trunk remained relatively stable in both conditions, with slight upward drift of a small magnitude but no statistically significant effects (Supplementary Figures 44 – 46). In contrast, significant time main effects indicated a decrease in leg ϕ over the measurement period (*p*<0.0001; Supplementary Figures 47 – 48).

### Body Fluids

No condition by time interactions were observed for body fluid outcomes. However, time main effects indicated drift over time was present in both conditions (*p* values of 0.0003 to <0.0001), with values of TBW and ICW decreasing over the measurement duration (Figure 3A – 3D). In contrast, no significant effects for ECW were observed (Figure 3E – 3F).

Segmental analysis indicated that decreases in total TBW and ICW were likely due to decreasing values in the arms and trunk rather than the legs (Supplementary Figures 49 – 58). Opposing segmental changes in ECW were observed, with ECW of the arms possibly decreasing (*p*-values of 0.01 to 0.001), ECW of the legs increasing (*p*<0.0001), and no change in trunk ECW (Supplementary Figures 59 – 63).

### Body Composition

The increase in BM after water consumption was solely detected as FM, with final values ~1.3 kg higher than baseline, on average (*p*<0.0001 for condition by time interaction; Figure 1C – 1D). FM drifted higher to a lesser degree in the control condition and was ~0.7 kg higher than baseline, on average, at the end of the measurement period. Greater increases in FM were observed in the water condition for the trunk and right leg (*p*-values of 0.0003 to <0.0001 for condition by time interactions), with significant time main effects but no interactions observed for the left leg and arms (Supplementary Figures 64 – 68). A condition by time interaction (*p*<0.0001) was also present for estimated visceral adipose tissue area, with the same trends as total FM. Specifically, VAT area increased over time in both conditions but increased to a greater extent in the water condition as compared to control (Supplementary Figure 69).

In contrast to FM, LBM did not differ between conditions and drifted lower throughout the measurement period in both conditions (*p*=0.0001 for time main effect; Figure 1E – 1F). At the final time point, LBM was approximately 0.8 kg lower than baseline in both conditions. Based on directionality of segmental LBM changes, decreases in total LBM in both conditions may be attributable to the arms and trunk regions (Supplementary Figures 70 – 74), while possible slight increases in leg LBM were observed. Total dry lean mass and estimated skeletal muscle mass demonstrated similar results to total LBM, with time main effects (*p*<0.0001 for both) indicating a downward drift in both conditions (Supplementary Figures 75 – 76).

BF% increased over time in both conditions but to a greater degree in the water condition (*p=*0.0001 for condition by time interaction; Supplementary Figure 77). Final BF% values were ~0.75% and ~1.5% higher than baseline, on average, in the control and water conditions, respectively.

## Discussion

The present investigation determined the effects of acute water ingestion on raw bioelectrical variables and subsequent estimates of body fluids and composition from a modern vertical bioimpedance analyzer, along with clarifying the effects of remaining in an upright body position for ~65 minutes. The broad findings were: 1) acute water ingestion increased Z and Xc of the arms, particularly at measurement frequencies ≤50 kHz, without clearly influencing the legs or trunk; 2) change over time or “drift” in numerous variables, irrespective of water ingestion and presumably due to maintaining an upright posture, was observed; these variables included leg Z and Xc, body fluids (TBW and ICW) and LBM, among other outcomes; 3) the increase in BM after water ingestion was detected exclusively as FM rather than body fluid or LBM; FM also drifted higher throughout the measurement period. While not fully discussed within the manuscript text, the supplemental materials present additional detail regarding the effects of water ingestion and time on a variety of raw bioimpedance and body composition variables estimated by the bioimpedance analyzer.

Bioimpedance is used to describe a wide variety of biological components, track physiological adaptations to lifestyle interventions, and inform evaluation of health and disease risk [1, 39, 40]. Although prior investigations have indicated a detrimental influence of unstandardized participant presentation, including the intake of food and fluids [10, 11, 12, 13, 14, 41], relatively few have examined water intake alone. However, the influence of water ingestion prior to assessments is potentially relevant as many investigations implement overnight fasts without restrictions on water intake or even recommend water intake in the hours prior to BIA assessments to promote euhydration.

Dixon *et al*. [16, 17] performed two experiments to determine the influence of fluid consumption on BIA variables. It was first demonstrated that foot-to-foot single-frequency BIA (Tanita TBF-300A) did not detect changes in Z or TBW in the 60 minutes following ingestion of 591 mL of water or a carbohydrate/electrolyte drink, although increases in BM and %BF estimates were observed [16]. Subsequently, a similar experiment was performed using an 8-point, hand-to-foot single-frequency analyzer (Tanita BC-418) [17]. Following ingestion of 591 mL of water, a 12-Ω increase in Z was observed, along with an increase in %BF of ~1%. However, the control condition with no fluid ingestion demonstrated a similar 11-Ω increase in Z and slight elevation of %BF of ~0.5%. In both investigations, participants sat between baseline and subsequent BIA assessments. It is worth noting that both BIA analyzers used in the studies of Dixon *et al*. have since been discontinued.

In the present investigation, which employed a modern, commercially available multi-frequency analyzer (InBody 770) and required participants to stand for the entire visit, the elevation of FM variables could have been driven both by the clear increase in BM detected by the scale component of the BIA analyzer and the differences in raw bioelectrical variables relative to the control condition. On average, FM was ~1.3 kg higher than baseline at the final assessment in the water condition, with ~0.9 kg of this increase occurring immediately after water ingestion. Meaningful upward drift in FM also occurred in the control condition, with final values ~0.7 kg higher than baseline at the final time point. While the magnitude of these errors is likely unimportant for one-time assessments, such as those used in epidemiological investigations and other cross-sectional settings, the implications of the artificial change in outcome variables may be greater when performing serial assessments over time. In this scenario, the magnitude of error could be non-negligible and either inflate or deflate the estimation of true changes depending on whether this error was present for the baseline evaluation, subsequent assessment, or both [15]. It should also be noted that these biologically induced errors, due to water intake and body fluid shifts, are notably larger than the TEM values of the MFBIA analyzer. Importantly, the present results for fluid volumes and body composition variables derived from raw bioimpedance may differ from effects that would be observed with other analyzers due to the device-specific manner in which these estimates are produced.

The clear drift in many variables over the course of 65 minutes of standing was presumably due to progressive fluid redistribution. The occurrence of dynamic fluid shifts upon changing body position has been established independent of bioimpedance techniques. Maw *et al*. [18] performed an informative investigation of postural changes on fluid volumes using simultaneous radionuclide dilution. While TBW remained constant throughout supine, seated, and standing postures, employed for 30 minutes each, blood volume was increased by 89 mL on average when supine and decreased by 406 mL (~6%) on average when standing, both relative to the seated position. In the standing position, the majority of volume shift occurred within the first 15 minutes and was attributable to an increase in interstitial fluid rather than expansion of ICW. These findings are consistent with other descriptions of the hemodynamic responses to the upright posture, which include a rapid redistribution of thoracic blood volume upon standing and a slower decline in plasma volume due to gravity-induced transcapillary diffusion [42].

In the present investigation, the general trends of increasing Z of the arms and decreasing Z of the legs, with little variation in the trunk, are consistent with the biophysical relationship between these variables and body fluids, as well as the expectation of fluid redistribution in the upright posture. While not definitively demonstrated, the directionality of changes in segmental fluid compartments also generally supports the redistribution of fluids from the upper to lower appendages with prolonged standing. From a practical standpoint, while apparent drift in some variables was observed throughout the measurement duration even without water ingestion, an ~15-minute period of upright rest prior to BIA assessments using vertical analyzers may help reduce unwanted variation in bioimpedance and variables derived from these values. For example, the greatest change in whole body ϕ – arguably the most used raw bioimpedance metric – was observed in first 15 minutes upright rest in the control condition.

Depending on the variable, the drift over time either accentuated or combatted the influence of water ingestion. For example, whole body ϕ displayed a clear drift of ~-0.1° on average within the first 15 minutes of standing in the control condition that was maintained throughout the visit. However, the ingestion of water apparently exerted an opposing effect, as ϕ values in the water condition did not change throughout the measurement duration. While the magnitude of change in ϕ observed in the control condition was relatively small compared to population-level SDs of ~0.5° to 1.1° [43, 44], it could be meaningful for longitudinal investigations attempting to detect changes in ϕ [5].

Importantly, the use of plain water ingestion as the physiological perturbation in the present investigation has several implications for BIA technology. A sufficient intake of water could affect body geometry by increasing volume and could also potentially decrease resistivity due to temporary dilution of ion concentrations. It has previously been demonstrated that bioelectrical variables are sensitive to changes in ion status [45].

Several investigations have described the influence of postural changes on fluid shifts using bioimpedance-derived variables; however, few have included serial assessments in the upright position, and standing durations have been relatively short (≤ 30 min) [22, 23, 46]. Gibson *et al*. [23] reported that, over the course of 30 minutes supine, bioimpedance spectroscopy ECW estimates decreased by 2.8%, ICW estimates increased by 2.5%, and no change in TBW (≤ 0.3%) was observed due to the opposing compartmental shifts. Conversely, when participants stood for 30 minutes, ECW estimates increased by only 0.8% with a 0.9% decrease in ICW. Several other investigations have collectively demonstrated an increase in whole-body Z when moving to the supine body position, with effects observed immediately and progressively increasing over at least four hours [19, 20, 21, 22, 23, 24].

Interestingly, some data indicate that following 60 minutes in the supine position, R normalizes to baseline values after only five minutes of standing [20]. Five minutes is also the duration of standing recommended in the product manual of the analyzer used in the present investigation. However, other data indicate that 30 minutes may be insufficient to achieve a steady state in fluid compartments estimated by bioimpedance [22]. It is not fully apparent if a true steady state was reached in the present investigation. While we observed no whole-body change in ECW (~0.4%), segmental evaluation indicated progressive declines in arm ECW (~1.9%) and increases in leg ECW (~1.8%). These findings are consistent with the idea that postural changes influence bioimpedance-derived variables due to fluid shifts in the extremities and subsequent alteration of limb volume, cross-sectional area, and muscle hydration [19, 47]. In contrast to the lack of change in whole-body ECW, slight decreases in whole-body ICW (~1.4%) and TBW (~1%) estimates were observed in the present study.

In conclusion, the present report provides data concerning two important methodological considerations for bioimpedance assessments. Acute water consumption altered some raw bioelectrical values and consistently elevated FM estimates without differentially influencing TBW, ECW, ICW, or LBM. Notable drift in most variables occurred during 65 minutes of upright posture, with some changes being clearly evident in the first 15 minutes of upright rest. As such, a period of 15 minutes of upright rest prior to assessments using vertical BIA analyzers may help reduce error, if consistently implemented. These findings have implications for pre-assessment standardization, methodological planning and reporting, and interpretation of bioimpedance-based metrics.

## Supplementary / e-repository

Supplementary figures:

https://github.com/joeb-files/2022_Tinsley

## Appendix

***P*-values for outcome variables**. Values produced from joint tests of linear mixed effects models. Bold values indicate statistical significance at the Bonferroni-adjusted alpha level of *p*<0.00057 (0.05/87 outcomes). Data accompanying these values are presented in Figures 1 – 3 and Supplementary Figures 1 – 77. *Abbreviations*: Xc – reactance; Z– impedance; ϕ – phase angle

**Table:**

	Condition	Time	Condition*Time
Body mass	**0.0003**	**<0.0001**	**<0.0001**
Total body water	0.06	**0.0003**	0.63
Intracellular water	0.08	**<0.0001**	0.36
Extracellular water	0.06	0.26	0.90
Dry lean mass	0.24	**<0.0001**	0.11
Fat mass	**<0.0001**	**<0.0001**	**<0.0001**
Lean body mass	0.09	**0.0001**	0.45
Skeletal muscle mass	0.07	**<0.0001**	0.42
Body fat percentage	**<0.0001**	**<0.0001**	**0.0001**
Right arm lean body mass	**0.0003**	0.002	0.49
Left arm lean body mass	0.02	0.01	0.54
Trunk lean body mass	0.01	0.002	0.55
Right leg lean body mass	0.76	**0.0001**	0.29
Left leg lean body mass	0.98	**<0.0001**	0.86
Right arm total body water	**0.0003**	0.001	0.37
Left arm total body water	0.02	0.01	0.43
Trunk total body water	0.01	0.002	0.63
Right leg total body water	0.76	**<0.0001**	0.18
Left leg total body water	0.95	**<0.0001**	0.92
Right arm intracellular water	0.001	0.003	0.49
Left arm intracellular water	0.02	0.02	0.60
Trunk intracellular water	0.01	**<0.0001**	0.72
Right leg intracellular water	0.91	0.54	0.04
Left leg intracellular water	0.95	0.11	0.28
Right arm extracellular water	**0.0002**	0.001	0.20
Left arm extracellular water	0.03	0.01	0.10
Trunk extracellular water	0.00	0.25	0.37
Right leg extracellular water	0.67	**<0.0001**	0.19
Left leg extracellular water	0.87	**<0.0001**	0.80
Right arm fat mass	0.00	**<0.0001**	0.01
Left arm fat mass	0.00	**<0.0001**	0.001
Trunk fat mass	**<0.0001**	**<0.0001**	**<0.0001**
Right leg fat mass	**<0.0001**	**<0.0001**	**0.0003**
Left leg fat mass	**<0.0001**	**<0.0001**	0.04
Visceral adipose tissue area	**0.0001**	**<0.0001**	**<0.0001**
Right arm Z at 1 kHz	**0.0002**	**<0.0001**	**<0.0001**
Left arm Z at 1 kHz	0.001	**<0.0001**	**<0.0001**
Trunk Z at 1 kHz	0.50	0.22	0.08
Right leg Z at 1 kHz	0.24	**<0.0001**	0.01
Left leg Z at 1 kHz	0.36	**<0.0001**	0.08
Right arm Z at 5 kHz	0.00	**<0.0001**	**<0.0001**
Left arm Z at 5 kHz	0.00	**<0.0001**	**<0.0001**
Trunk Z at 5 kHz	0.34	0.09	0.12
Right leg Z at 5 kHz	0.25	**<0.0001**	0.03
Left leg Z at 5 kHz	0.34	**<0.0001**	0.02
Right arm Z at 50 kHz	**0.0002**	**0.0002**	**<0.0001**
Left arm Z at 50 kHz	0.002	0.001	**0.0001**
Trunk Z at 50 kHz	0.24	0.16	0.12
Right leg Z at 50 kHz	0.25	**<0.0001**	0.05
Left leg Z at 50 kHz	0.36	**<0.0001**	0.01
Right arm Z at 250 kHz	**0.0001**	0.001	**0.0004**
Left arm Z at 250 kHz	0.001	**0.0004**	0.001
Trunk Z at 250 kHz	0.30	0.47	0.13
Right leg Z at 250 kHz	0.24	**<0.0001**	0.08
Left leg Z at 250 kHz	0.33	**<0.0001**	0.002
Right arm Z at 500 kHz	**0.0001**	0.0008	0.001
Left arm Z at 500 kHz	0.002	**0.0001**	0.002
Trunk Z at 500 kHz	0.22	0.67	0.11
Right leg Z at 500 kHz	0.22	**<0.0001**	0.08
Left leg Z at 500 kHz	0.25	**<0.0001**	0.001
Right arm Z at 1000 kHz	**0.0001**	0.001	0.001
Left arm Z at 1000 kHz	0.00	**<0.0001**	0.01
Trunk Z at 1000 kHz	0.39	0.89	0.09
Right leg Z at 1000 kHz	0.18	**<0.0001**	0.05
Left leg Z at 1000 kHz	0.33	**<0.0001**	0.004
Right arm Xc at 5 kHz	**0.0004**	**<0.0001**	**<0.0001**
Left arm Xc at 5 kHz	0.001	**<0.0001**	**0.0002**
Trunk Xc at 5 kHz	0.51	**0.0004**	0.21
Right leg Xc at 5 kHz	0.22	**<0.0001**	0.05
Left leg Xc at 5 kHz	0.28	**<0.0001**	0.29
Right arm Xc at 50 kHz	0.00	**<0.0001**	**<0.0001**
Left arm Xc at 50 kHz	0.01	**<0.0001**	0.001
Trunk Xc at 50 kHz	0.77	0.03	0.18
Right leg Xc at 50 kHz	0.29	**<0.0001**	0.01
Left leg Xc at 50 kHz	0.33	**<0.0001**	0.01
Right arm Xc at 250 kHz	0.07	0.71	0.005
Left arm Xc at 250 kHz	0.001	0.02	**0.0001**
Trunk Xc at 250 kHz	0.30	0.46	0.14
Right leg Xc at 250 kHz	0.25	**<0.0001**	0.01
Left leg Xc at 250 kHz	0.17	**<0.0001**	0.01
Right arm ϕ	0.29	0.01	0.002
Left arm ϕ	0.45	0.002	0.17
Trunk ϕ	0.62	0.12	0.13
Right leg ϕ	0.41	**<0.0001**	0.02
Left leg ϕ	0.41	**<0.0001**	0.01
Whole body ϕ	0.33	0.01	**0.0004**
Urine specific gravity	0.03	**0.004**	**0.0004**
